# Coumarin-transition metal complexes with biological activity: current trends and perspectives

**DOI:** 10.3389/fchem.2024.1342772

**Published:** 2024-02-12

**Authors:** Lozan T. Todorov, Irena P. Kostova

**Affiliations:** Department of Chemistry, Faculty of Pharmacy, Medical University–Sofia, Sofia, Bulgaria

**Keywords:** coumarin, transition metals, coordination compounds, biological activity, current trends

## Abstract

Coumarin (2H-1-benzopyran-2-one) presents the fundamental structure of an enormous class of biologically active compounds of natural, semi-synthetic, and synthetic origin. Extensive efforts are continually being put into the research and development of coumarin derivatives with medicinal properties by the broad scientific community. Transition metal coordination compounds with potential biological activity are a “hot topic” in the modern search for novel drugs. Complexation with transition metals can enhance the physiological effect of a molecule, modify its safety profile, and even imbue it with novel attributes of interest in the fields of medicine and pharmacy. The present review aims to inform the reader of the latest developments in the search for coumarin transition metal complexes with biological activity, their potential applications, and structure-activity relationships, where such can be elucidated. Each section of the present review addresses a certain kind of biological activity (antiproliferative, antioxidant, antimicrobial, etc.), explores the most recent discoveries in the field, and, at the same time, tries to offer useful perspectives for potential future investigations.

## 1 Introduction

**FIGURE 1:**
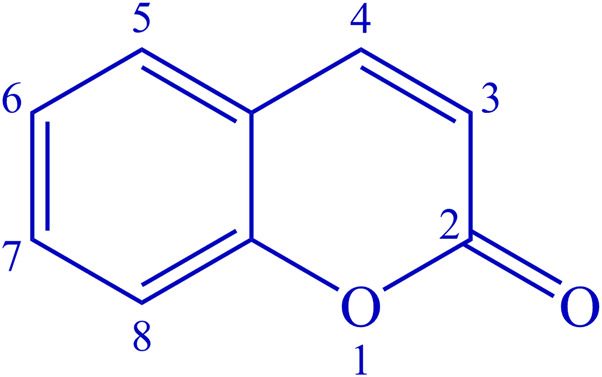
Structure of the coumarin heterocycle.

Coumarins, derivatives of 2H-1-benzopyran-2-one (Figure 1), are a large class of oxygen-bearing heterocyclic substances, ubiquitously present in plants as secondary metabolites (Robe et al., 2021). In nature, they can be found combined with sugars as glycosides (Bartnik and Facey, 2024). More than 1,300 different natural coumarins have been isolated from the seeds, fruits, flowers, roots, and stems of hundreds of plant species (Matos et al., 2015), serving as components of defense mechanisms against herbivores and contamination from microorganisms.

Coumarins can be substituted at various sites of their base structure. One of the vast number of possible substitution patterns of the coumarin scaffold serves as the basis for their numerous physiological activities: anticoagulant (Kumar et al., 2022), anticancer (Thakur et al., 2015; Rawat and Reddy, 2022), antimicrobial (Al-Majedy et al., 2017; Cheke et al., 2022), anti-inflammatory (Bansal et al., 2013; Grover and Jachak, 2015), and neuroprotective (Epifano et al., 2009; Matos et al., 2020) activities, etc. In recent years, increasing efforts have been focused on the potential utilization of the optical properties of coumarins in the field of medicine. Coumarins are highly fluorescent chromophores (Katerinopoulos, 2004). They are easy to synthesize, chemically stable, and are characterized by generally high quantum yields (Budzák et al., 2016). Novel therapeutic approaches, such as photodynamic therapy (PDT), involve the application of photosensitizing transition metal complexes with optically active ligands (Teo et al., 2016). The coumarin scaffold is increasingly being utilized in the search for novel photodynamic agents. Some important biogenic elements, serving a variety of functions in living organisms (Kostova, 2023), are transition metals. Their variable oxidation state, hence the ability to participate in redox reactions, make them important bioactive agents (Grass et al., 2011; Bagchi et al., 2015). Platinum complexes, for example, are well known anticancer drugs (Kelland, 2007). Metals like mercury, silver, and gold have been applied in medicine for millennia. It is the authors’ observation that in recent years more and more transition metal coordination complexes are being tested as potential therapeutic agents. The major driving forces behind this process seem to be the attempts to overcome microbial and cancer drug resistance (Huang et al., 2021). Metal coordination with biologically active ligands can result in enhanced effect (Todorov et al., 2023), reduced toxicity (Kongot et al., 2019), and even completely novel mechanisms of action (Imberti et al., 2020). Coumarins are a class of compounds that combine a wide spectrum of biological activities with excellent chelating properties, making them ideal candidates for the synthesis of novel complexes with potential therapeutic utility. A number of detailed reviews on transition metal coumarin complexes with biological activity have been published over the previous decade (Balcıoğlu et al., 2020; Balewski et al., 2021; Patil et al., 2022). The present review aims to inform the reader on the latest developments in the field over the period 2020-2023. Though coumarins and their derivatives tend to have multiple biological activities at the same time, the authors have tentatively classified the complexes presented herein in the following manner:

- Coumarin complexes with antimicrobial activity
- Coumarin complexes with anticancer activity
- Coumarin complexes as photodynamic and photochemotherapeutic agents
- Coumarin complexes as enzyme inhibitors and antihaemolytic agents

Antioxidant activity is prominent in coumarins and their coordination compounds. The authors would like to direct the reader to a recently published review article (Todorov et al., 2023) that deals with this aspect of their biological activity in detail.

## 2 Coumarin complexes with antimicrobial activity

**FIGURE 2:**
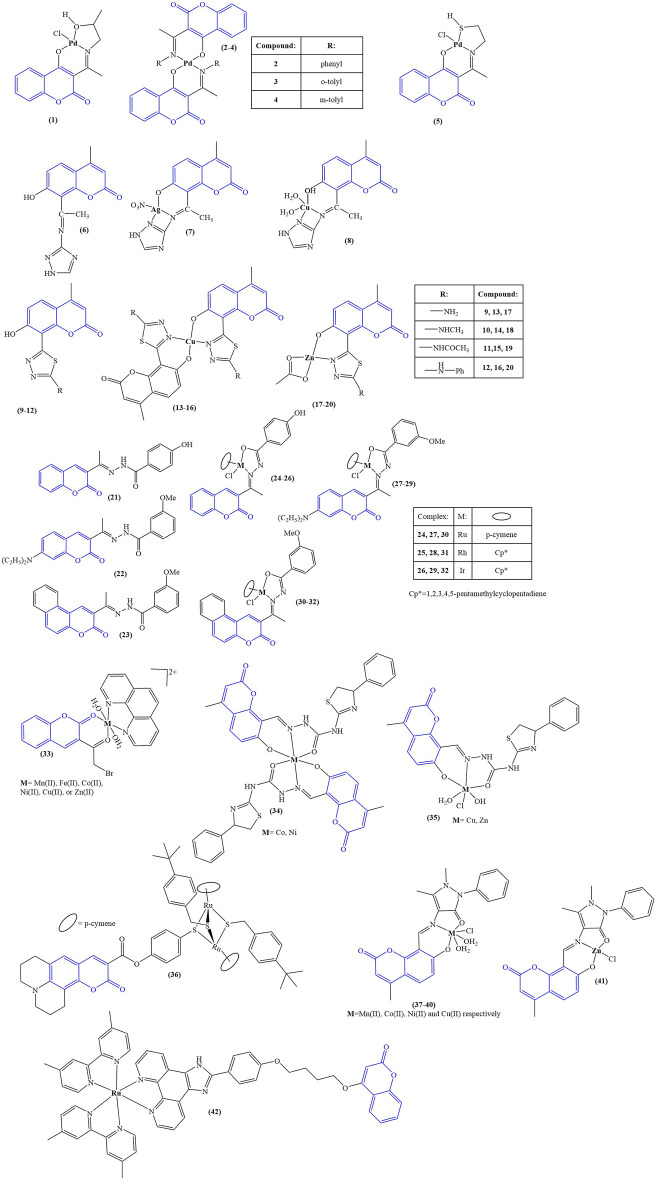
Coumarin complexes with antimicrobial activity.

The structures of the coumarin complexes with antimicrobial activity discussed below are presented in Figure 2.

Aldovic and coworkers synthesized several coumarin-derived ligands and coordinated them with Pd(II) (compounds 1–5) (Avdović et al., 2019). All tested compounds manifested moderate to low antimicrobial activity. Few of them acted selectively. Ligand 1 and its corresponding compound 1 manifested minimal inhibitory concentration (MIC) of 125 μg/mL and 62.5 μg/mL respectively toward *Aspergillus flavus* ATCC, comparable to the value for the standard substance fluconazole with MIC = 62.5 μg/mL. The same compounds had MICs of 62.5 μg/mL when tested against *Bacillus subtilis* IP 5832 and *Bacillus cereus*, compared to less than 2.0 μg/mL exhibited by the standard substance doxycycline. Generally, coordination of the ligands with Pd(II) tended to increase antibacterial activity.

Abdel-Kader and coworkers synthesized a Shiff base chelator from 8-acetyl-7-hydroxy-4-methylcoumarin and 3-amino-1,2,4-triazole (Abdel-Kader et al., 2021). The novel compound (compound 6) was coordinated with silver and copper (compounds 7 and 8). Both ligand and complexes were tested *in vitro* for antimicrobial activity against gram-positive bacteria (*B. subtilis, S. aureus, S. faecalis*), gram-negative bacteria (*E. coli, N gonorrhoaeae, P. aeruginosa*), and fungi (*A. flavus, C. albicans*). Inhibition zone diameters were measured. All compounds were found to be inactive against the fungal strains. In terms of antibacterial activity, the compounds’ activity was compared to the positive control ampicillin. The inhibition zone diameter of ampicillin varied between 21 and 28 mm for all bacterial strains. The same zone was 9–10 mm for the ligand. Coordination with silver increased activity (12–13 mm). Copper coordination did not improve activity in most cases (9–11 mm), while in some (*S. faecalis, N. gonorrhoaeae*), activity was completely negated (0 mm). The authors of the paper proposed that the observed lower activity of the copper complex was due to its lower lipophilicity, compared to the ligand and its silver counterpart.

Karcz and coworkers synthesized series of coumarin-thiadiazole hybrids (compounds 9–12) and coordinated them with Cu(II) (compounds 13–16) and Zn(II) (compounds 17–20) (Karcz et al., 2021). The novel ligands were found to be bidentate, coordinating with Zn(II) in 1:1 molar ratio and with Cu(II) in 2:1 molar ratio. The novel compounds were tested against several types of microbes–*E. coli, P. aeruginosa, S. aureus* (Gram-negative), *S. epidermidis* ATCC12228*,* and *S. epidermidis* ATCC35984 (Gram-positive). The ligand (12) and complexes (16,20), bearing a phenylamino moiety, tended to be the most active antibacterial agents (MIC = 0.9–3.12 mg/mL against all microbial strains). This activity was significantly weaker than the positive controls chloramphenicol, gentamicin, and kanamycin, whose MIC values were in the 10^–3^ to 10^–2^ mg/mL order of magnitude. Gram-positive bacteria were more sensitive to exposure, compared to gram-negative. Complexation with both Cu(II) and Zn(II) decreased activity, with MICs increasing by 100%–200% compared to the free ligands. This observed negative effect was stronger in the copper complexes compared to their zinc counterparts, even though the former bear two ligand molecules, while the latter bear only one.

Nongpiur and coworkers synthesized a number of half-sandwich platinum group metal complexes, containing coumarin-N-acylhydrazone hybrid ligands (Nongpiur et al., 2021). Each ligand (compounds 21–23) was coordinated with ruthenium, rhodium, or iridium (compounds 24–32). Antimicrobial activity was measured against *S. aureus, B. thuringiensis* (gram-positive), *E. coli,* and *P. aeruginosa* (gram-negative). Results were expressed as zone of inhibition at 5 mg/mL (agar well diffusion study). Kanamycin was used as a positive control. None of the compounds (ligands and complexes) manifested any activity against the gram-negative strains. None of the ligands had noticeable antibacterial activity, the exception being compound 23 with zone of inhibition 16 ± 1 mm against *B. thuringiensis*. Compound 23s complexes were found to be inactive. Compound 22 was inactive, but its complexes manifested antibacterial activity against the gram-positive strains. The most active complex, compound 28, had zones of inhibition of 19 ± 1 mm (*S. aureus*) and 16 ± 1 mm (*B. thuringiensis*), compared to 21 ± 1 mm and 20 ± 1 mm for kanamycin. Overall, complexation with Ru, Rh, and Ir seemed to increase antibacterial activity. Compounds 21–32 underwent a DPPH assay (0.004% DPPH in methanol, 1 mg/mL tested compound, 30 min cultivation in darkness), which was applied to test potential antioxidant activity. Ascorbic acid (1 mg/mL) was used as a positive control. Results were presented as % of the activity of ascorbic acid. Out of the three ligands, 21 manifested highest activity (39.7% ± 3.9%), most probably due to the phenolic hydroxyl moiety. The most active compound was its Ir complex 26 (85.0% ± .04%). The Ir complex of ligand 22 (compound 29) also manifested significant activity of 50.6% ± 1.7%. Overall, complexation with any of the metals seemed to increase DPPH-scavenging. Ir had a positive effect on ligands 21 and 22, while with 23 the effect of Ir was negative. Coordinating Ru with 23 caused an increase of scavenging from 21.7% ± 0.5% to 37.6% ± 1.6%. DNA binding of 22 and its complexes 27–29 was tested with salmon milt DNA. Only with compound 29 did the authors observe interaction with DNA, attributing it to DNA groove binding.

A series of complexes were synthesized, incorporating 3-(bromoacetyl)coumarin, 1,10-phenanthroline as a second ligand and a variety of transition metal ions–Mn(II), Fe(II), Co(II), Ni(II), Cu(II), and Zn(II) (compound 33) (El-Shwiniy et al., 2020). The complexes were tested for antimicrobial activity against bacteria (*S. aureus, L. monocytogens, B. cereus, A. baumanii*) and fungi (*A. niger, A. terreus*). 1 mL 1 × 10^−3^ M solutions of the tested compounds were incubated 20 h at 37 C for bacteria and 7 days at 30 C for fungi. Results were presented as zone of inhibition diameters in mm. Both ligands and all complexes were found to be inactive against the fungal strains. Against *S. aureus*, most active were compound 33-Co(II) and compound 33-Zn(II) (inhibition diameters of 30 ± 0.5 mm and 35 ± 1.3 mm respectively). The positive controls moxifloxacin and ciprofloxacin had zones of inhibition of 22 ± 1.7 and 27 ± 0.4 mm. *L. monocytogens* was most sensitive to compound 33-Ni(II) and compound 33-Cu(II) (29 ± 0.41 mm and 30 ± 0.2 mm inhibition diameter, compared to 14 ± 0.1 mm and 18 ± 0.4 mm for the positive controls). Compound 33-Co(II), compound 33-Mn(II), and compound 33-Zn(II) were most active against *B. cereus* (35 ± 1.25 mm, 34 ± 1.1 mm, and 34 ± 1.6 mm inhibition zone, compared to 22 ± 0.3 mm and 23 ± 0.1 mm for the positive controls). *A. baumantii* was most sensitive to compound 33-Ni(II) and compound 33-Zn(II) (inhibition zones 33 ± 1.9 mm and 30 ± 0.55 mm, compared to 16 ± 1.2 and 14 ± 1.2 for moxifloxacin and ciprofloxacin). That study revealed that while the complexes in most cases were more potent antibacterial agents, compared to the positive controls, the ligands by themselves were also very active, particularly 1,10-phenanthroline (inhibition zones varied between 20 ± 0.04 mm and 30 ± 0.33 mm against the bacterial strains). The observed antibacterial effect was indeed sometimes improved by complexation with the investigated transition metals, however, in some cases antimicrobial activity was either unchanged, or actually diminished as a result of complexation.

A number of octahedral complexes of Cu(II), Co(II), Ni(II), and Zn(II) with a Shiff base ligand, derived from 8-formyl-7-hydroxy-4-methylcoumarin, were synthesized (Yernale and Mathada, 2020). Co(II) and Ni(II) coordinated two ligand molecules (compound 34), and Cu(II) and Zn(II) one molecule (compound 35). The ligand and the complexes were tested against a panel of bacterial (*S. aureus* MTCC3160, *B. subtilis* MTCC 736, *E. coli* MTCC 46, *S. typhi* MTCC 98) and fungal strains (*C. albicans* MTCC227, *C. oxysporum* MTCC1777, *A. flavus* MTCC 1883, *A. niger* MTCC 1881) by disk diffusion and well diffusion methods respectively. The compounds were tested at concentrations 12.5, 25, 50, 75, and 100 μg/mL. Activities were presented as minimum inhibitory concentration, defined in this case as the minimum tested concentration with no visible growth. Against the bacterial strains the MIC of the ligand itself was 75 μg/mL, with the exception of *S. aureus* (MIC = 50 μg/mL). Complexation with the metal ions caused an increase in activity with MICs dropping to 25 μg/mL. The Zn(II) complex manifested lesser potency with MIC = 25 μg/mL against *S. aureus* and MIC = 50 μg/mL against the other bacterial strains. The positive control gentamicin completely suppressed bacterial growth at 12.5 μg/mL. Complexation also increased antifungal activity–MIC decreased from 50–75 μg/mL (ligand) to 25–50 μg/mL (complexes). The positive control fluconazole had MIC = 12.5 μg/mL. Cleavage of coiled plasmid DNA pBR322 was observed in presence of all compounds. Brine shrimp lethality bioassay was used to assess cytotoxicity. The ligand had LD50 = 2.262 × 10^−4^ M/mL. Coordinating it with metal ions increased toxicity. Most active were the Co(II) and Ni(II) complexes, LD50 = 1.106 × 10^−4^ M/mL and 1.112 × 10^−4^ M/mL respectively. The activity of the Cu(II) and Zn(II) was close to that of the ligand, suggesting that in this model system the number of coordinated ligands play a major role in cytotoxicity.

Desiatkina and coworkers synthesized coumarin-tagged trithiolato-bridged ruthenium (II) arene complexes (Desiatkina et al., 2020). Compounds, bearing a linker between the coumarin moiety and the di-ruthenium scaffold, suppressed the proliferation of *Toxoplasma gondii* at 1 μM concentration but also impaired human foreskin fibroblast (HFF) cell viability. The type of substituent, attached to the thiolate component of the complexes, significantly impacted antiparasitic activity, *tert*-butyl causing an increase in activity, compared to trifluoromethyl. The most active complex (compound 36) manifested IC_50_ = 0.105 nM against *T. gondii*. At the same time, at 2.5 μM, HFF viability was reduced to 28% of the negative control. At the IC_50_ molarity, Concavalin A-induced T-cell proliferation was suppressed (69% of negative control), and bacterial lipopolysaccharide induced B-cell proliferation. The compound did not seem to impact the metabolic activity in T cells and B cells. Mitochondrial function in *T. gondii* tachyzoites, infecting HFF monolayers, was significantly impaired after 24 h treatment with compound 36 at IC_50_ concentration. After 48h, tachyzoites were completely devoid of mitochondria.

Mujahid and coworkers synthesized a series of Cu(II) and Zn(II) complexes with previously reported 2-(2-oxo-2H-chromene-substituted-yl)oxy acetic acids as ligands (Mujahid et al., 2023). The complexes were tested for antimicrobial activity against methicillin-resistant *S. Aureus*, *P. Aeruginosa*, and *C. Albicans*. Vancomycin was used as a positive control for the bacterial study and Amphotericin-B for the *C. Albicans* test. Previous study of the ligands demonstrated no activity against these pathogens. Their Ag(I) complexes, also previously reported (Mujahid et al., 2016), had a moderate to weak antibacterial effect and in some cases had significant antifungal activity, comparable to amphotericin-B. In contrast, the novel zinc and copper complexes, incorporating the same ligands, did not manifest any antibacterial activity. The authors of the study proposed that the difference between the coordination mode of the Ag(I) complexes on one hand and the Cu(II) and Zn(II) on the other may be the reason for these results. As the metal ion seems to exert the antimicrobial activity, the coordination compounds reported in the study may not release their metal ion, unlike their Ag(I) counterparts.

A coumarin-bearing tridentate ligand was synthesized and coordinated with several different bivalent metal ions - Mn(II), Co(II), Ni(II), Cu(II), and Zn(II)–compounds 37–41 respectively (Sunitha et al., 2023). Their antifungal activity was tested against *C. albicans* and *A. niger*. Results were presented as zone of inhibition diameter with clotrimazole as the positive control. The ligand itself suppressed fungal growth only mildly with zone of inhibition of 5 mm against both strains. Complexation improved activity, the best results being yielded by the copper complex (*C. albicans -* 14 mm, *A. niger*–9 mm). Zones of inhibition of clotrimazole were 20 mm and 19 mm respectively. When tested against gram-negative *E. coli*, the ligand had the most prominent antibacterial behavior (MIC = 0.113 μg/mL), followed by the Ni(II) complex (MIC = 0.118 μg/mL). The same complex was the most active against *S. aureus* (MIC = 0.110 μg/mL) with the Mn(II)-bearing compound 37 being second (MIC = 0.173 μg/mL). The ligand in this case was not as potent as against *E. coli* (MIC = 0.232 μg/mL).

Huang and coworkers (Huang et al., 2023) synthesized a coumarin-bearing ligand and, together with a series of ancillary ligands, coordinated it with Ru(II). The ancillary ligands were 2,2′-bipyridine and 2,2′-bipyridine disubstituted at positions 4 and 4′ with methyl (compound 42), methoxy, and *tert*-butyl substituents. The novel complexes were tested against gram-negative *E. coli* and *P. aeruginosa* and were found to be inactive. Compound 42 manifested antibacterial activity against gram-negative *S. aureus* (MIC = 1.56 μg/mL). Further studies showed it could interact with phospholipids in the bacterial membrane, generating reactive oxygen species, consequently impairing membrane integrity.

A summary of the data on the activity of the most active compounds, described in this section, can be viewed in Table 1.

**TABLE 1:** Summary of the most active antimicrobial compounds presented herein.

Compound	*Microbial strain*	Activity–tested compound	Activity - control substance (if tested)
1 and its Pd(II) complex		MIC	Gentamicin (MIC)
	*Bacillus subtilis IP 5832*	62.5 μg/mL	<2.0 μg/mL
	*Bacillus cereus*	62.5 μg/mL	<2.0 μg/mL
			Fluconazole (MIC)
	*Aspergillus flavus ATCC*	62.5–125 μg/mL	62.5 μg/mL
7		Inhibition zone	*Ampicillin*
	Gram-positive (*B. subtilis, S.aureus, S. faecalis*), gram-negative bacteria (*E. coli, N gonorrhoaeae, P. aeruginosa*)	12–13 mm	21–28 mm
	*A. flavus, C. albicans*	Inactive (0 mm)	
12,16,20	Gram-negative (*E.coli, P. aeruginosa, S. aureus),* Gram-positive (*S. epidermidis ATCC12228, S. epidermidis ATCC35984)*	MIC	Chloramphenicol, gentamicin, and kanamycin (MIC)
		0.3–3.12 mg/mL	10^–3^ to 10^–2^ mg/mL
28		Inhibition zone	Kanamycin
	*S. aureus*	19 ± 1 mm	21 ± 1 mm
	*B. thuringiensis*	16 ± 1 mm	20 ± 1
Zn(II)-33		Inhibition zone	Ciprofloxacin
	*S. aureus*	35 ± 1.3 mm	27 ± 0.4 mm
	*B. cereus*	34 ± 1.6 mm	23 ± 0.1 mm
	*A. baumantii*	30 ± 0.55 mm	14 ± 1.2 mm
34,35		MIC^1^	Gentamicin
	*S. aureus* MTCC3160*, B.subtilis* MTCC 736*, E. coli* MTCC 46*, S. typhi* MTCC 98	25–50 μg/mL	12.5 μg/mL
	*C. albicans* MTCC227, *C. oxysporum* MTCC1777, *A. flavus* MTCC 1883, *A. niger* MTCC1881	25–50 μg/mL	12.5 μg/mL
36	*T. gondii*	IC_50_: 0.105 nM	No control tested
37		MIC	Control tested was not named
	*S. aureus*	0.173 μg/mL	
39	*S. aureus*	0.110 μg/mL	
	*E. coli*	0.118 μg/mL	
		Zone of inhibition	Clotrimazole
40	*C. albicans*	14 mm	20 mm
	*A. niger*	9 mm	19 mm
42	*S. aureus*	MIC	No positive control tested
		1.56 μg/mL	

1 MIC, defined in this case as the minimum tested concentration with no visible growth.

## 3 Coumarin complexes with anticancer activity

**FIGURE 3:**
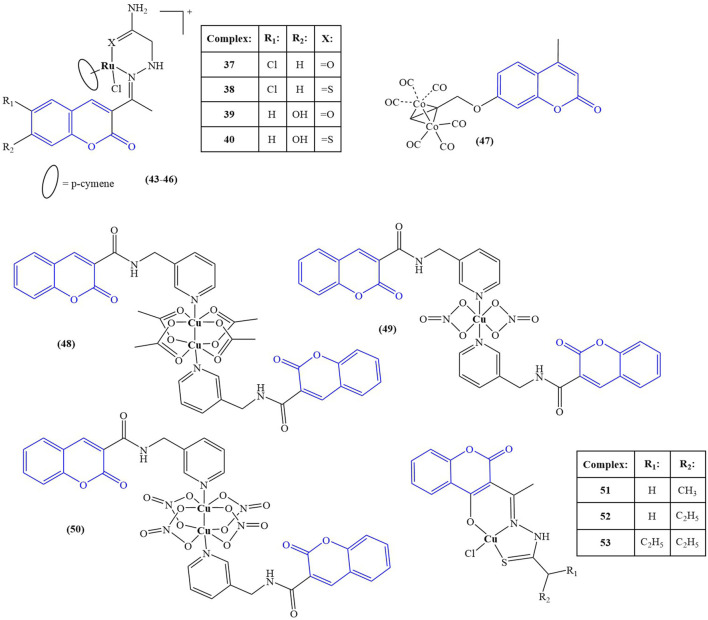
Coumarin complexes with anticancer activity.

The structures of the coumarin complexes with anticancer activity discussed below are presented in Figure 3.

A series of Ru(II) complexes (compounds 43–46), containing coumarin-based nitrogen and oxygen/sulphur donor chelators, were synthesized and tested for *in vitro* cytotoxicity (Kalaiarasi et al., 2022) against A549, MCF-7 cancer cell lines and normal HUVEC cells. Cisplatin was used as a positive control. The cell lines were incubated at 37°C for 24 h, followed by 48 h treatment with the tested compounds. All ligands manifested low toxicity (IC_50_ > 100 μM) against both normal and cancer cells. Complexation with Ru(II) resulted in significant cytotoxic effect. Against A549, the complexes had IC_50_ between 8.32 ± 1.58 μM and 13.54 ± 1.98 μM. Against MCF-7, IC_50_s varied between 6.61 ± 2.29 μM and 11.72 ± 2.49 μM. Cisplatin had IC_50_ = 27.38 ± 1.54 μM (A549) and IC_50_ = 43.72 ± 2.81 μM (MCF-7). The electron-donating OH-group at position 7 of the coumarin ring and coordination of Ru(II) to sulphur, instead of oxygen improved cytotoxicity–the most potent compound 46 incorporated both components in its structure. All complexes had low toxicity against normal HUVEC cells (IC_50_ > 200 μM). Acridine orange-ethidinium bromide assay was applied as a qualitative and quantitative method to detect apoptosis. A549 and MCF-7 cells were treated for 24 h with the respective IC_50_ concentrations of the complexes. Compound 46 caused the highest apoptosis ratio of close to 40% for both cancer cell lines–more than twice that of the positive control cisplatin. Hoechst 33,258 staining assay after 48 h treatment with the IC_50_ concentrations was used to detect morphological changes in cell nuclei. In treated MCF-7 and A549 cells, a significant increase in chromatin condensation and nuclear shrinkage was noted, with compound 46 being the most potent of the complexes.

Compounds 6 and 7 (see chapter 2) (Abdel-Kader et al., 2021) were tested for antiproliferative activity against MCF-7 breast cancer and HCT-116 colon cancer cell lines, using cisplatin as a positive control. The ligand was found to possess very low activity (IC_50_ > 325.40 μM for both cell lines). Coordination with silver dramatically improved activity against both strains (IC_50_ = 23.29 μM). The control substance cisplatin had IC50 = 5.64 μM and 17.73 μM against MCF-7 and HCT-116 cell lines.

Liu and coworkers synthesized seven complexes, incorporating coumarin ligands and carbonyl cobalt with the aim of producing potential anticancer agents with the ability to release carbon monoxide (Liu et al., 2022). Antiproliferative activity of the novel compounds was tested against Hep2G, HeLa, and MDA-MB-231 tumor cell lines (MTT assay, 10 μM–150 μM concentrations, 24 h incubation, 5-fluorouracil (5-FU) as a positive control). The most potent complex (compound 47) had IC_50_ values of 93.68 ± 21.40 μM (HeLa), 48.15 ± 4.58 μM (HepG2), and 34.98 ± 7.57 μM (MDA-MB-231)–significantly lower that the positive control 5-FU (IC_50_ = 115.87 ± 6.44, 205.25 ± 18.69, and 83.04 ± 2.99 μM for HeLa, HepG2, and MDA-MB-231 respectively). The authors attributed the promising anticancer activity of this compound to the presence of the electron-donating methyl group in the pyran ring. Substituting this group with fluorine-bearing, electron-withdrawing functionalities significantly negated the antiproliferative effect. Compound 47 was further tested for its impact on cell viability of the same cancer strains. At 50 μM, cell viability was 37.67% (Hep2G), 61.49% (HeLa), and 45.89% (MDA-MB-231). Cell viability of MDA-MB-231 in the presence of μM 5-FU was higher, compared to **47**. The impact of **47** on ROS generation was measured on MDA-MB-231 treated for 24 h with 40 μM and 80 μM of the compound with the help of dichlorodihydrofluorescin acetate. A significant, concentration-dependent accumulation of ROS was observed, with the authors clarifying that the observed effect was not due to fluorescence by the coumarin structure itself. Mitochondrial membrane potential was also impaired, and western blotting revealed upregulation of Bax, downregulation of Bcl-2 and activation of caspase-3. The authors concluded that apoptosis induction in MDA-MB-231 by compound 47 could be attributed to the mitochondrial dysfunction signal pathway. Molecular docking revealed that this compound could be inserted into the active pocket of Bcl-2.

Lu *et al.* synthesized coumarin-3-formyl-(3-aminomethylpyridine) in order to utilize it as a ligand to generate three different Cu(II) complexes - a binuclear Cu(II) acetate complex, a mononuclear Cu(II) nitrate complex, and a binuclear Cu(II) nitrate complex (Lu et al., 2023)–compounds 48–50 respectively. The ligand and the complexes were tested against HeLa, HepG2, MCF-7, and A549 human cancer cell lines and the normal HUVEC cell line using the MTT assay. The ligand was found to be inactive, with IC_50_ > 150 μM. HeLa cells most sensitive to compound 48 (IC_50_ = 29.33 ± 0.33 μM), compared to cisplatin (IC_50_ = 3.03 ± 0.39 μM). It was inactive against all other cell strains. Compounds 49 and 50 showed moderate activity against all cancer strains with the exception of compound 49 which was more cytotoxic against MCF-7 than cisplatin (IC_50_ = 2.86 ± 0.08 μM *versus* 9.07 ± 0.10 μM). Another positive attribute of that complex was its lack of toxicity against normal HUVEC cells (IC_50_ > 150 μM), compared to cisplatin (IC_50_ > 0.58 ± 0.05 μM). Experiments with herring sperm DNA showed that compound 49 intercalates with the stacked DNA base pairs.

Compounds 37–41 (see chapter 2) (Sunitha et al., 2023) were also investigated for antiproliferative activity against MCF-7 and K-562 cancer cell lines utilizing the Sulforhodamine B assay with Adriamycin as the positive control. The ligand was found to be inactive within the tested range of concentrations (IC_50_ > 80 μg/mL). Complexation with the selected transition metal ions caused moderate cytotoxicity against both cell lines with the exception of the Co(II)-bearing compound 38 which had a potent effect, comparable to the positive control (IC_50_ < 10 μg/mL for both the complex and Adriamycin against both cell lines). Additional DNA cleavage study with plasmid pUC-18 DNA showed moderate cleaving activity for the ligand and the complexes.

Shreshtha *et al.* (Shrestha et al., 2024) synthesized a series of coumarin-based thiosemicarbazones and coordinated them with Cu(II)–compounds 51–53. The complexes and their respective ligands were investigated for antiproliferative activity toward MCF-7 and MDA-MB-231 cell lines. Complexation tended to decrease cytotoxicity against MCF-7. IC_50_ values of the ligands were between 12.94 μg/mL and 18.36 μg/mL. The corresponding Cu(II) complexes had IC_50_ between 20.80 μg/mL (compound 53) and 23.70 μg/mL (compound 52). In the case of MDA-MB-231, results were “mixed”. Compound 51 was found to be inactive, while its corresponding ligand had IC_50_ = 66.65 μg/mL. Compound 46 was significantly more potent than the corresponding ligand (IC_50_ = 47.23 μg/mL *versus* 80.21 μg/mL). The activities of compound 53 and its ligand were about the same (IC_50_ = 48.29 μg/mL and 42.18 μg/mL respectively). Western blot analysis showed that compound 47 downregulated the antiapoptotic Bcl2, while upregulating the proapoptotic Bax protein.

A summary of the data on the activity of the most active compounds, described in this section, can be viewed in Table 2.

**TABLE 2:** Summary of the most active compounds with anticancer activity presented herein.

Compound	Cancer cell lines	Activity–tested compound	Activity - control substance (if tested)
7		IC_50_	Cisplatin
	MCF-7	23.29 μM	5.64 μM
	HCT-116	23.29 μM	17.73 μM
38		IC_50_	Adriamycin
	MCF-7	<10 μg/mL	<10 μg/mL
	K-562	<10 μg/mL	<10 μg/mL
46		IC_50_	Cisplatin
	A549	8.32 ± 1.58 μM	27.38 ± 1.54 μM
	MCF-7	6.61 ± 2.29 μM	43.72 ± 2.81 μM
47		IC_50_	5-Fluorouracil
	HeLa	93.68 ± 21.40 μM	115.87 ± 6.44 μM
	HepG2	48.15 ± 4.58 μM	205.25 ± 18.69 μM
	MDA-MB-231	34.98 ± 7.57 μM	83.04 ± 2.99 μM
48		IC_50_	Cisplatin
	HeLa	29.33 ± 0.33 μM	3.03 ± 0.39 μM
	HepG5	>150 μM	6.06 ± 0.44 μM
	MCF-7	>150 μM	9.07 ± 0.10 μM
	A549	>150 μM	1.68 ± 0.05 μM
	HUVEC	>150 μM	0.58 ± 0.05 μM
49	MCF-7	2.86 ± 0.08 μM	9.07 ± 0.10 μM
	HUVEC	>150 μM	0.58 ± 0.05 μM
53		IC_50_	No control tested
	MCF-7	20.80 μg/mL	
	MDA-MB-231	48.29 μg/mL	

## 4 Coumarin complexes as photodynamic and photochemotherapeutic agents

**FIGURE 4:**
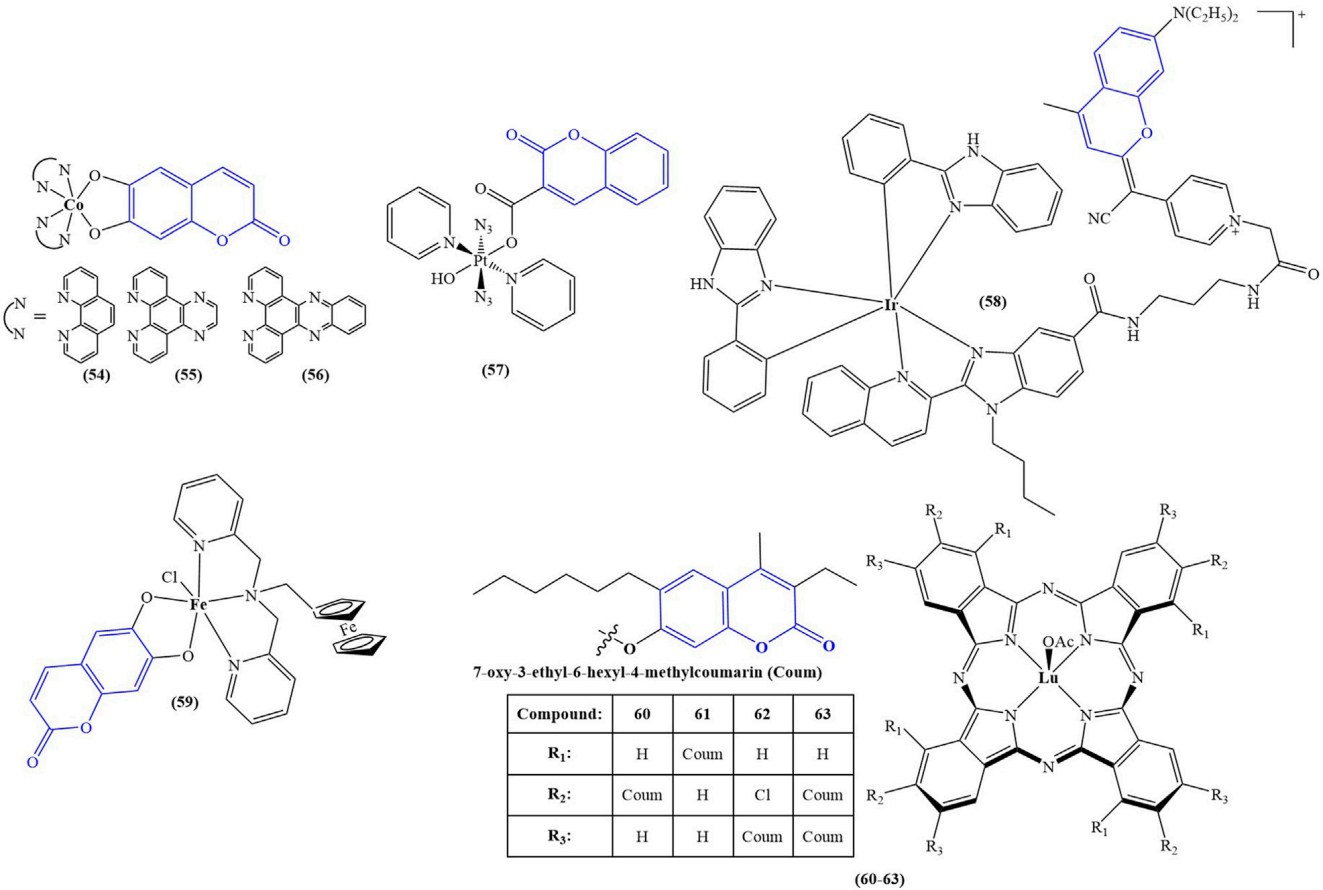
Coumarin complexes with potential application as photodynamic and photochemotherapeutic agents.

The structures of the optically active coumarin complexes with potential medicinal applications discussed below are presented in Figure 4.

Sarkar and coworkers (Sarkar et al., 2021) synthesized a series of mixed-ligand cobalt (III) complexes, utilizing a N,N-donor phenanthroline base and O,O-donor dianionic ligand, derived from esculetin (6,7-dihydroxycoumarin). The structures of the complexes are presented as compounds 54–56. In terms of potential biological activity, several types of assays were carried out. Photocytotoxicity in the presence of low-energy visible light (400–700 nm, 10 J/cm^-2^) was evaluated against HeLa and MCF-7 cancer cell lines. In dark conditions, none of the complexes manifested significant toxicity (IC_50_ > 50 μM). In light conditions, the complex, bearing the photoactive ligand (compound 56), showed very low IC_50_ = 1.09 ± 0.1 μM (MCF-7) and IC_50_ = 1.6 ± 0.1 μM (HeLa), with a phototoxicity index (PI = IC_50_(dark)/IC_50_(light)) of 31.7 and 45.9 respectively. Activity increased in the following order: 56 > 55>54. For comparison, the commercial photodynamic agent Photofrin had IC_50_ = 4.3 ± 0.2 and PI = 9.5. These results speak for excellent photodynamic activity, accompanied by low toxicity in dark conditions–desirable qualities for any photodynamic agent. Exchanging the esculetin ligand with catechol decreased toxicity in light conditions and consequently PI values. Cellular localization study of complex **56** revealed that it tends to accumulate in mitochondria. It was observed to induce early features of apoptosis in HeLa cells when irradiated. Tests on ROS generation revealed this complex does not cause singlet oxygen generation in light conditions but generates superoxide radicals instead. It bound significantly with ct-DNA. DNA melting study and viscosity measurements suggested interaction with DNA surface, followed by groove binding. Compounds 54, 55, and 56 were not DNA cleavage-active in the dark. In light conditions, DNA photocleavage increased 56 > 55>54.

A platinum (IV) prodrug, containing a coumarin ligand, was tested as a potential agent for photoactivated chemotherapy (PACT) (compound 57) (Bolitho et al., 2021). Unlike PDT, PACT does not require oxygen. Light is used to chemically modify the structure of a prodrug, thus helping release the active substance intracellularly. The authors had previously reported diazido Pt (IV) complexes that are inactive in dark conditions but form cytotoxic Pt (II) and azidyl radicals when irradiated with visible light. The authors propose that the coumarin component of **compound 57** could improve anticancer activity by acting as a light-harvesting antenna, through its intrinsic antiproliferative activity and by improving overall lipophilicity. The complex was tested against PC3 cancer cells–2 h exposure in dark conditions, or 1 h exposure, followed by 1 h irradiation with 465 nm light. In darkness, the complex had IC_50_ > 100 μM. Upon photoactivation, IC_50_ dropped to 6.48 ± 0.84 μM. The activity of the positive control (cisplatin) was low in both light and dark conditions (IC_50_ > 100 μM). Cells, treated with low concentrations of 015 (0.25x IC_50_) in light conditions showed noticeable morphological deviations from the intreated controls–cytoplasmic vacuolization (a process associated with cell death) and membrane-blebbing. Treatment with IC_50_ concentration of 6.5 μM, followed by irradiation with blue light, caused significant cellular damage, and multiple cytoplasmic vacuoles were observed, the nuclei seemed to be damaged, mitochondria and lipids were difficult to identify in the cytoplasm, and severe blebbing of the plasma membrane was noted. In addition, PC3 cells were allowed to recover for 2 h in complex-free medium after 1 h treatment with 6.5 μM, followed by 1 h irradiation. They appeared to be significantly recovered, compared to those treated with no recovery. The presence of the coumarin component significantly increased accumulation of platinum in whole PC3 cancer cells, compared to a previously studied complex, bearing a hydroxyl group, instead of the coumarin structure. It was confirmed that irradiation increased the amount of Pt (II) and decreased Pt (IV) in the cells, compared to treatment in dark conditions.

A cyclometallated iridium (III) complex (compound 58) was conjugated to a far red emitting coumarin with the aim of producing a photosensitizer for PDT (Novohradsky et al., 2021). The novel complex was tested against prostate cancer stem cells, tumorspheres, formed from surface marker CD151-positive and CD151-negative phenotypes of DU145 cells. Tests were performed in both dark and light (420 nm, 28 J cm^-2^, 30 min) conditions by way of CellTiter-Glo 3D cell viability assay. The complex manifested strong antiproliferative activity against both phenotypes in light conditions (IC_50_ = 5.7 ± 0.2 μM and IC_50_ = 5.9 ± 0.6 μM) and low toxicity in dark (IC_50_ > 100 μM). RealTime-Glo annexin apoptosis assay revealed a marked increase of apoptosis in tumorspheres from the CD151-positive subtype (1.5 h incubation in dark, followed by 0.5 h irradiation with 28 J cm^-2^, blue light). The complex increased intracellular calcium influx, stimulated autophagy, and raised ROS generation, the effect being markedly stronger in light conditions, compared to cells treated in darkness.

A tumor-selective ferrocenyl iron (III) coumarin conjugate (compound 59) was synthesized as a potential photochemotherapeutic agent (Sarkar et al., 2020). Ferrocene-conjugated dipicolylamine and esculetin were chosen as ligands. Confocal microscopy showed cytosolic localization of the complex after 4 h incubation. The nucleus staining dye Hoechst 33,258 and the mitochondria staining dye Mitotracker Deep Red showed prominent accumulation in mitochondria and no accumulation in the nucleus. Photocytotoxicity was evaluated in HeLa, MCF-7, and HaCaT cancer cell lines under low energy visible light (400–700 nm, 10 J/cm^2^) and red light (600–720 nm, 50 J/cm^2^). MTT assay revealed the complex had low toxicity in dark conditions (IC_50_ > 50 μM), but after visible light irradiation its cytotoxicity rose dramatically (IC_50_ between 3.2 ± 0.8 and 7.4 ± 1.0 μM against the cancer cell lines). Even in low intensity red light, this compound performed as a powerful antiproliferative agent (IC_50_ between 8.8 ± 1.3 and 15.3 ± 1.7 μM). Healthy MCF-10A cells were also tested with the compound. In visible light IC_50_ = 31.6 ± 2.7 μM, and in red light IC_50_ > 42 μM.

Ozdemir and coworkers synthesized a series of lutetium (III) phthalocyanine-coumarin dyads (compounds 60–63) as potential PDT sensitizers (Ozdemir et al., 2021). The complexes were tested for singlet oxygen generation and photodegradation under light irradiation. Compounds 60, 61, and 63 showed relatively high singlet oxygen quantum yields of 0.84, 0.83, and 0.90 respectively. In addition, the complexes were tested for radical scavenging ability. ABTS scavenging was most prominent with compounds 62 and 63 - IC_50_ = 120.34 and 188.73 mM Trolox/mg respectively (higher value is better). In butylhydroquinone (BHT), the standard compound had IC_50_ = 52.63 mM Trolox/mg. The activity of the other two compounds was estimated as 20 times weaker. Ferric Reducing Antioxidant Power assay showed compounds 61 and 63 to be the most potent (IC_50_ = 0.375 and 0.356 mM Fe^2+^/mg respectively), about 2–3 times more potent than compounds 60 and 62 but less active than the standard BHT ((IC_50_ = 1.1 mM Fe^2+^/mg). Compounds 60 and 62 were also the stronger copper reducing agents (IC_50_ = 2.040 and 1.775 mM trolox respectively), about an order of magnitude more potent than 60 and 62. Their activity was weaker than that of vitamin C (IC_50_ = 2.70 mM trolox).

A summary of the data on the activity of the most active compounds, described in this section, can be viewed in Table 3.

**TABLE 3:** Summary of the most active photodynamic/photochemotherapeutic agents presented herein.

Compound	Cancer cell lines	Activity–tested compound	Activity - control substance (if tested)
56		IC_50_(dark)	Photofrin (visible light): 4.3 ± 0.2 μM
	HeLa	>50 μM	
	MCF-7	>50 μM	
		IC_50_(visible light)	
	HeLa	1.6 ± 0.1 μM	
	MCF-7	1.09 ± 0.1 μM	
57	PC3	IC_50_(dark)	Cisplatin
		>100 μM	IC_50_ > 100 μM (dark and light)
		IC_50_(light)	
		6.48 ± 0.84 μM	
58		IC_50_(dark)	No control tested
	DU154 (CD151-positive)	>100 μM	
	DU154 (CD151-negative)	>100 μM	
		IC_50_(light)	
	DU154 (CD151-positive)	5.7 ± 0.2 μM	
	DU154 (CD151-negative)	5.9 ± 0.6 μM	
59		IC50(dark)	Esculetin: Photofrin
	MCF-7	>50	13.6 ± 1.2
	HeLa	>50	20.3 ± 1.4 >41
	HaCaT	>50	11.8 ± 1.7
	MCF-10A	>50	>42
		IC50(visible light)	
	MCF-7	3.2 ± 0.8 μM	11.4 ± 1.3
	HeLa	7.4 ± 1.0	18.2 ± 1.5
	HaCaT	7.4 ± 1.0	15.4 ± 1.6
	MCF-10A	31.6 ± 2.7	15.4 ± 1.6
		IC50(red light)	
	MCF-7	8.8 ± 1.3	4.3 ± 0.2
	HeLa	15.3 ± 1.1	
	HaCaT	11.8 ± 1.7	
	MCF-10A	>42	

## 5 Coumarin complexes as enzyme inhibitors and antihaemolytic agents

**FIGURE 5:**
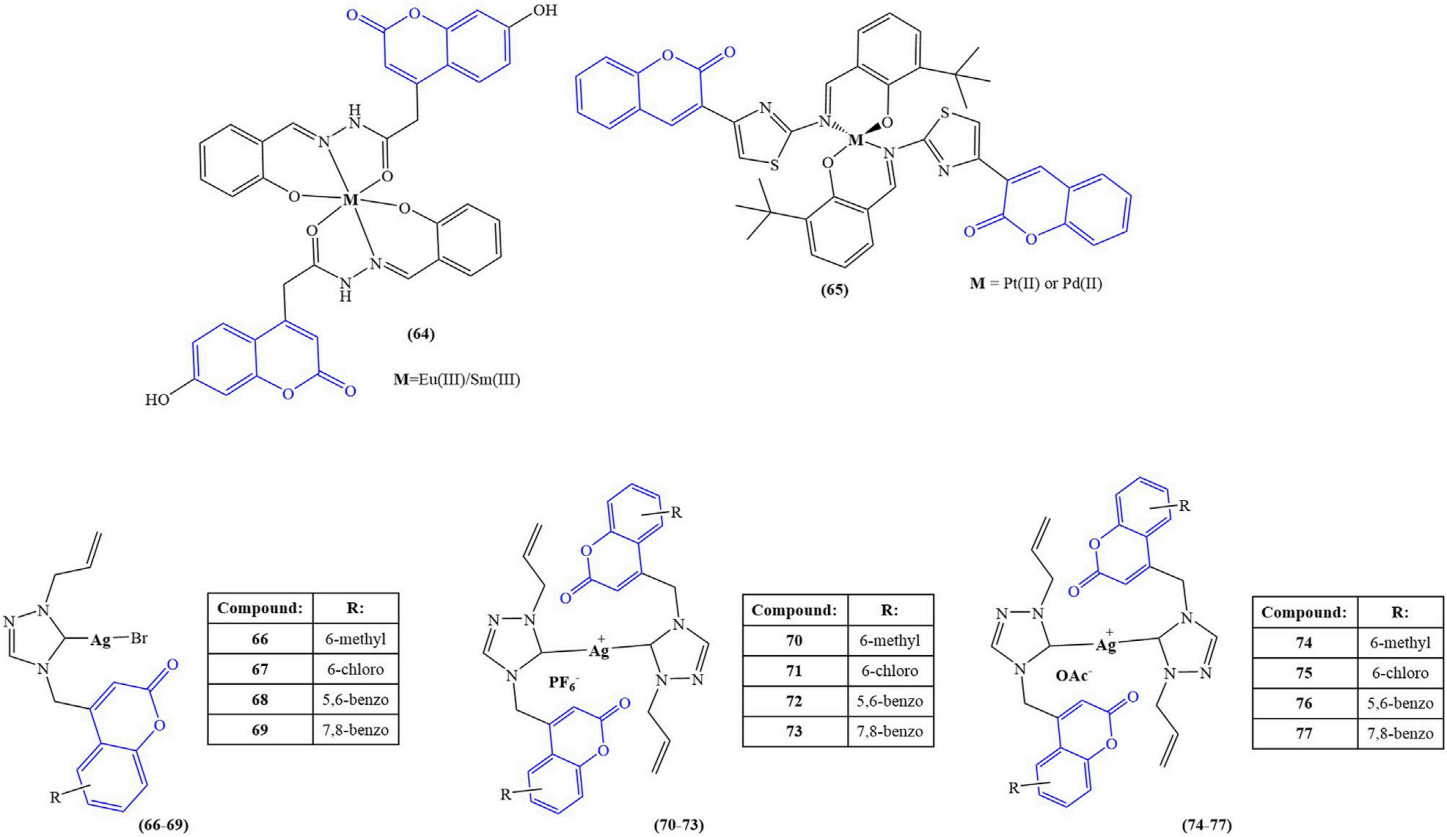
Coumarin complexes with potential application as enzyme inhibitors and antihaemolytic agents.

The structures of the coumarin complexes with potential enzyme inhibitory properties are presented in Figure 5.

Elsenety and coworkers synthesized a novel coumarin-based tridentate ligand, 4-(2-hydroxy benzylidene acetohyrazide)-7-hydroxy coumarin, and coordinated it with the lanthanide ions Eu(III) and Sm(III) (compound 64) (Elsenety et al., 2020). Fluorescent studies show characteristic luminescence of both lanthanide ions, revealing a typical “antenna effect” behavior on the side of the chelator. Molecular docking with the active sites of xanthine oxidase chain C revealed the presence of H-donor interactions between the nitrogen atoms of the ligand and the GLN 1194 amino acid residue.

Milenkovic *et al* performed molecular docking study of the ligand 3-(1-m-toluidinoethylidene)-chromane-2,4-dione and its Pd(II) complex (compound 4) (Milenković et al., 2020). The ligand and the Pd(II) complex were investigated with the aim to elucidate the binding mode to the cycline-dependent kinase 2 (CDK2) receptors (AutoDock software). CDK2 participates in DNA replication during the G1/S phase and is crucial for the progression of the S phase. Inhibition of CD2 could be a target for potential chemotherapeutic agents. The docking study revealed that the free ligand has better potential to inhibit the receptor, compared to the complex.

Acetylcholinesterase (AChE) activity of compounds 9–20 (Karcz et al., 2021) was tested by measuring absorption at 412 nm for 30 min at 37°C. The most notable changes were observed within the first 5 min. Tacrine was used as a control. Unlike the antibacterial assays, complexation did not cause as much of a change in IC_50_ values. The most active was compound 11, bearing an amide group (IC_50_ = 0.181 ± 0.0123 μM) and its Cu(II) and Zn(II) complexes 15 and 19 (IC_50_ = 0.174 ± 0.0181°μM and 0.184 ± 0.0069 μM respectively). The standard compound, tacrine, had IC_50_ = 0.053 ± 0.0036 μM. The authors proposed that decreased water solubility of the complexes, compared to the free ligands, may be involved in the observed lowering of anti-AChE activity.

Sahin and coworkers synthesized a Schiff base-coumarin hybrid and coordinated it with Pd(II) or Pt (II) (compound 65) (Şahin et al., 2022). The novel compounds were tested for inhibitory activity toward AChE, butyrylcholinesterase (BChE), and pancreatic cholesterol esterase (CEase). Pyridostigmine was used as a positive control for AChE and BChE. The ligand manifested activity against AChE (IC_50_ = 22 μM *versus* 23 μM for pyridostigmine). It had zero BChE inhibitory activity, compared to IC_50_ = 138 μM for pyridostigmine. Complexation with both metals increased inhibition, the observed effect being stronger in the Pt (II) complex (IC_50_ = 12 μM and 23 μM for AChE and BChE respectively). In terms of CEase inhibition, the ligand had IC_50_ = 24 μM. Coordination with Pd(II) significantly decreased inhibition (IC_50_ = 57 μM), while Pt (II) slightly increased inhibition (IC_50_ = 21 μM). Molecular docking (Molegro Virtual Docker–Molegro A 2019) suggested that, unlike the ligand and its Pt (II) counterpart, the Pd(II) complex weakly binds to amino acids and interacts poorly with amino acid residues that are important to enzyme catalytic functions.

Geetha and coworkers investigated a series of coumarin-incorporated 1,2,4-triazole-derived Ag(I) N-heterocyclic carbenes for antioxidant and antihaemolytic activity (Ge et al., 2020). The structure of the ligands was presented as compounds 54–61. Three types of complexes were synthesized: mono-NHC-coordinated silver bromides (compounds 66–69), bis-NHC-coordinated hexafluorophosphates (compounds 70–73), and bis-NHC-coordinated silver acetate (compounds 74–77). DPPH assay revealed that silver nitrate and the ligands themselves manifest no radical-scavenging activity. The complexes themselves tended to scavenge DPPH. Complexes bearing methyl substituent at sixth position of the ligand’s coumarin core structure behaved as the best scavengers. Bis-NHC-coordinated hexafluorophosphates (compounds 70–73) had the most significant effect (IC_50_ between 61 ± 14 μM and 131 ± 7 μM). The activity of the mono-NHC-silver bromides (compounds 66–69) was lower (IC_50_ between 136 ± 6 μM and 210 ± 6 μM). Interestingly, the bis-NHC silver acetate complexes (compounds 74–77) were even weaker scavengers of DPPH (IC_50_ between 165 ± 6 μM and 224 ± 4 μM). The positive control gallic acid had IC_50_ = 22 ± 5 μM. Antihaemolytic activity was evaluated at 100 μM concentrations and presented as the percentage lysis of human red blood cells. The positive control (triton X) showed 95.62% ± 0.27% lysis. The ligands showed very low haemolytic activity (less than 1%). Similar to the DPPH assay, the 6-methylcoumarin-bearing complexes performed better than their respective counterparts. Percentage lysis of red blood cells increased as follows: bis-NHC-coordinated hexafluorophosphates (% lysis between 2.32 ± 0.05 and 9.47 ± 0.38), mono-NHC-coordinated silver bromides (percentage lysis between 21.53 ± 0.32 and 51.30 ± 0.11), and bis-NHC-coordinated silver acetates (percentage lysis between 25.27 ± 0.32 and 46.91 ± 0.48). The authors concluded that bis-NHC coordinated silver hexafluorophosphates performed better than the other two classes of complexes synthesized both as DPPH scavengers and as antihaemolytic agents, suggesting further study of the compounds as potential anticancer and antimicrobial agents. A series of bis-NHC silver complexes, with similar ligands bearing a 2,6-dimethylphenyl substituent attached to the 1,2,4-triazole heterocycle, underwent the same testing (Geetha et al., 2020) in order to observe the effect of the counterion (PF_6_^−^, Br^−^, or acetate) on DPPH scavenging and haemolysis. All complexes bearing bromide counterions manifested DPPH-scavenging activity. Hexafluorophosphate and acetate counterions seemed to eliminate scavenging in some complexes. Acetate counterions seemed to increase haemolysis–the complexes with 5,6-benzo- and 7,8-benzo-substituted coumarins as ligands manifested a percentage lysis of red blood cells as high as 67.3% ± 1.19% and 32.71% ± 1.55%.

A summary of the data on the activity of the most active compounds, described in this section, can be viewed in Table 4.

**TABLE 4:** Summary of the most active enzyme inhibitors and antihaemolytic agents presented herein.

Compound	Biological activity	Assay performed
4	CDK2 inhibition	Molecular docking
11	AChE inhibitor	Tacrine (control)
	IC_50_ = 0.181 ± 0.0123 μM	IC_50_ = 0.053 ± 0.0036 μM
64	Interaction with xanthine oxidase	Molecular docking
Pt(II)-65	AChE and BChE inhibition	Pyridostigmine (control)
	IC_50_ = 12 μM (AChE) and 23 μM (BChE)	IC_50_ = 22 μM (AChE) and 138 μM (BChE)
70–73	Antihaemolytic antioxidants	Triton X (control)
	% lysis between 2.32% ± 0.05% and 9.47% ± 0.38%	% lysis 95.62% ± 0.27%

## 6 Discussion and conclusion

Natural and synthetic coumarins are a topic of intense research and discussion in the fields of medicine and pharmacy. A brief, surface online search on publications since 2020 yields dozens of reviews and hundreds, maybe thousands, of original research articles, encompassing a large variety of structural “subtypes”, pharmacological activities and diverse mechanisms of action. Conversely, published articles on coumarin-bearing supramolecular compounds with biological activity seem to be somewhat sparse. The authors have highlighted several previous review articles over the past decade (Peng et al., 2013; Pereira et al., 2018; Balcıoğlu et al., 2020; Balewski et al., 2021; Patil et al., 2022) that deal with that subject. Based on their reporting as well as the data presented herein, it seems that until relatively recently, investigative efforts in this area were few and far between and only started to gain some momentum during the past decade. Recent lifting of pandemic restrictions has probably contributed to intensification in the frequency of publications. After reviewing available literature, the authors have formulated some tentative conclusions and recommendations:

- Metal coordination tends to improve biological activity. This would be in agreement Overtone’s concept of cell permeability - the cellular membrane tends to favor the passage of hydrophobic molecules. Chelation theory suggests partial sharing of the positive charge of the transition metal ion with the donor groups, combined with π-delocalization within the chelate ring. As a result, lipophilicity and, hence, membrane permeation and biological activity tend to increase. As demonstrated by (Mujahid et al., 2023), there are additional factors at play - if biological activity is realized by the metal ion, its particular coordination mode determines whether it is “released” to exhibit its effect.
- In terms of “preferred” transition metal ions, researchers seem to focus on “safe bets” such as Pt (II/IV) and Pd(II) (antiproliferative agents) and Cu(II) and Zn(II) (antimicrobial chelates). Such experimentation is quite important, as these are metal ions with proven physiological effects. On the other hand, there are many other biologically active transition metal ions to explore. Gold and silver are prominent antiproliferative and antimicrobial agents. Ruthenium-based complexes are widely researched as potential anticancer drugs (Lee et al., 2020) and photodynamic/photochemotherapeutic compounds (Lu et al., 2022). Carbon monoxide-releasing carbonyl-cobalt (Gong et al., 2016; Kongot et al., 2019; Jana et al., 2023) and iridium (Gothe et al., 2016; De Palo et al., 2021) also seem to occur more and more frequently in the development efforts of new antiproliferative agents. Lanthanides are characterized by low toxicities (Evans, 2013; Gonzalez et al., 2014), intrinsic biological activity (Teo et al., 2016), and fluorescent properties (Li et al., 2020). The large number of biologically active transition metal ions combined with the relatively few publications on biologically active coumarin complexes offer a promising avenue for future research.
- The coumarin scaffold is an obvious choice for synthesis of biologically active chelating agents due to its numerous intrinsic physiological effects and significant potential for a wide variety of substitution patterns. Some successful efforts have been made (Kharadi and Patel, 2009; Kharadi, 2011) to produce supramolecular compounds that incorporate coumarins together with drug molecules aiming for a specific therapeutic effect. Rational design of novel ligands, bearing specific substituents with a pre-defined target (e.g., a tumor-specific membrane receptor) would help “focus” physiologically active metal ions on tissues where pathologies are developing while alleviating systemic toxicities. Another approach, observed in photodynamic therapy, involves systemic administration of a non-toxic photosensitizer that is locally “activated” under irradiation. Coumarins are known photosensitizers (Kasperkiewicz et al., 2016). Complexes bearing a coumarin ligand and another type of photoactive ligand (e.g., dipyridophenazine) tend to significantly improve phototoxicity of a complex while maintaining low toxicity in dark conditions. Conjugating coumarins with photoactive compounds seems to yield ligands with improved photodynamic properties.

Transition metal complexes are continually “gaining ground” in the fields of medicine and pharmacy. Extensive research efforts are invested in “traditional” applications of compounds such as cytostatics (da Silva et al., 2022) in radiotherapy (Gill and Vallis, 2019) and immunosuppressants (Song et al., 2023) to name a few. Complexation of coumarins is being intensively investigated in the field of microbial infection treatment. Coumarin compounds are known for their anticancer properties (Kostova, 2007; Thakur et al., 2015), however novel research of complexes with a “direct” anticancer effect are few and far between. What is noteworthy is that the photoactive core of the coumarin structure is gaining more and more popularity in the rapidly developing fields of photodynamic therapy and photochemotherapy. Promising results with coumarin-bearing Pt (IV) prodrugs for PACT demonstrate the excellent potential for major discoveries in these areas in the search for antineoplastic dugs with improved effectiveness, localized action, and an enhanced safety profile.
